# Microbial Fuel Cell Based on Nitrogen-Fixing *Rhizobium anhuiense* Bacteria

**DOI:** 10.3390/bios12020113

**Published:** 2022-02-11

**Authors:** Rokas Žalnėravičius, Algimantas Paškevičius, Urtė Samukaitė-Bubnienė, Simonas Ramanavičius, Monika Vilkienė, Ieva Mockevičienė, Arūnas Ramanavičius

**Affiliations:** 1 Centre for Physical Sciences and Technology, Sauletekio Av. 3, LT-10257 Vilnius, Lithuania; rokas.zalneravicius@ftmc.lt (R.Ž.); urte.samukaite-bubniene@chf.vu.lt (U.S.-B.); simonas.ramanavicius@ftmc.lt (S.R.); 2 Laboratory of Biodeterioration Research, Nature Research Centre, Akademijos 2, LT-08412 Vilnius, Lithuania; algimantas.paskevicius@gamtc.lt; 3 Department of Physical Chemistry, Faculty of Chemistry and Geosciences, Institute of Chemistry, Vilnius University, Naugarduko Str. 24, LT-03225 Vilnius, Lithuania; 4 Lithuanian Research Centre for Agriculture and Forestry, Instituto Av.1, Akademija, LT-58344 Kedainiai, Lithuania; monika.vilkiene@lammc.lt (M.V.); ieva.mockeviciene@lammc.lt (I.M.)

**Keywords:** nitrogen-fixing bacteria, *Rhizobium anhuiense*, menadione, microbial fuel cells

## Abstract

In this study, the nitrogen-fixing, Gram-negative soil bacteria *Rhizobium anhuiense* was successfully utilized as the main biocatalyst in a bacteria-based microbial fuel cell (MFC) device. This research investigates the double-chambered, H-type *R. anhuiense*-based MFC that was operated in modified Norris medium (pH = 7) under ambient conditions using potassium ferricyanide as an electron acceptor in the cathodic compartment. The designed MFC exhibited an open-circuit voltage (OCV) of 635 mV and a power output of 1.07 mW m^−2^ with its maximum power registered at 245 mV. These values were further enhanced by re-feeding the anode bath with 25 mM glucose, which has been utilized herein as the main carbon source. This substrate addition led to better performance of the constructed MFC with a power output of 2.59 mW m^−2^ estimated at an operating voltage of 281 mV. The *R. anhuiense*-based MFC was further developed by improving the charge transfer through the bacterial cell membrane by applying 2-methyl-1,4-naphthoquinone (menadione, MD) as a soluble redox mediator. The MD-mediated MFC device showed better performance, resulting in a slightly higher OCV value of 683 mV and an almost five-fold increase in power density to 4.93 mW cm^−2^. The influence of different concentrations of MD on the viability of *R. anhuiense* bacteria was investigated by estimating the optical density at 600 nm (OD_600_) and comparing the obtained results with the control aliquot. The results show that lower concentrations of MD, ranging from 1 to 10 μM, can be successfully used in an anode compartment in which *R. anhuiense* bacteria cells remain viable and act as a main biocatalyst for MFC applications.

## 1. Introduction

In order to meet the growing demand for human food, the agriculture industry is intensifying production by new technologies, some of which involve the excessive use of nitrogen with other elemental fertilizers and alter chemical products. According to Robertson and Vitousek [1], the global application of nitrogen fertilizers has increased by more than ten times in the last 50 years [2,3]. Although adding chemical nitrogen to agricultural systems has major benefits, there are numerous unpleasant environmental impacts. Recently, some studies have revealed that the use of nitrogen in agriculture is one of the main triggers for coastal zone eutrophication processes [4]. This process leads to hypoxia in the coastal zone and other surface water bodies. Algae blooms are also triggered by nitrogen (N) uptake from agricultural land [5,6]. Therefore, intensive agricultural systems emit reactive nitrogen-based gases, particularly ammonia and various nitrogen oxides, which act as powerful greenhouse gases in the troposphere [7,8,9].

As an alternative for chemical nitrogen fertilizers, soil bacteria could be used, which can fix atmospheric nitrogen. They occur either as free-living soil bacteria (e.g., *Azotobacter*, *Clostridium pasteurianum*) or in interaction with the roots of leguminous plants (e.g., *Rhizobium*, *Bradyrhizobium*) [10,11,12,13]. This alternative is more environmentally friendly and has several positive aspects. For instance, soil bacteria increase the biodiversity of soil organisms as well as stimulate biogeochemical cycles [14]. All these aspects lead to better soil health. The agronomic approach for these bacteria has been widely analyzed and used in practice [15,16,17], and it is hypothesized that they could provide power to microbial fuel cells, and, after this process, return to the soil ecosystem and act symbiotically with legumes for atmospheric N_2_ fixation. However, there is a lack of information about the use of this group of bacteria for microbial fuel cells and their potential to produce electrical power.

At the beginning of the nineteenth century, the first article was published about electricity that was produced by bacteria. The main research object was *Saccharomyces* or bacteria and their metabolic pathways [18]. For the next hundred years, this capability was tested/applied only in the laboratory. Moreover, in the last decade, researchers focused on “green” renewable energy under growing energy requirements and climate change. One research area for the promising generation of green energy is microbial fuel cells. A fuel cell is usually defined as a cell that converts chemical energy into electrical energy without any direct combustion [19,20]. Several different types of microbial fuel cells were reported during the last decade. We can group them according to the kind of energy production: benthic microbial fuel cells (BMFC) [21,22]; photosynthetic microbial fuel cells (PhMFCs) [23,24,25]; plant microbial fuel cells (PMFC) [26].

Bacterial species that have the ability to transfer electrons extracellularly are referred as exoelectrogens [27,28]. Several lists of bacteria consortiums that can be used in the generation of electricity are provided in Table 1. In all five reference lists, soil bacteria are included. Plant microbial fuel cell operation is based on the interaction of plant roots and microbes in the rhizosphere [26]. *Rhizobium* bacteria are classified as gram-negative and rod-shaped cells. Rhizobia–legume symbiosis is a well-documented example of symbiosis. Plants secrete flavonoids (pisatin, genistein) into the rhizosphere (active plant root zone) that activate rhizobial nod genes via the transcriptional activator NodD. Nod gene expression leads to the synthesis of the bacterial chemical signal, Nod factor, a lipochitin oligosaccharide. The Nod factor binds to specific plant kinases initiating a signalling pathway leading to root hair curling and trapping of rhizobia [29,30]. Microbial fuel cells employed by *Rhizobium* bacteria could provide a market for green energy. However, there is a lack of information on the design of MFC based on *Rhizobium* species bacteria.

Electroactive bacteria strains are important for power generation in MFC devices. In order to enhance the performance of MFC, many recent studies have been focused on the chemical and genetic modifications of microorganisms [34]. Luo et al. [35] reported the additional treatment of *K. rhizophila* bacteria with lysozyme, which accelerats electron transfer about 1.75 times. However, chemical modification usually brings some disadvantages, such as reduced microorganism viability and long-term stability, thus making the species more susceptible to the environmental biota. Genetic engineering has a significant impact, increasing the performance of MFCs via the modification of biocatalysts cells. Nandy et al. [36] showed that genetically “improved” *E. coli* cells through cloning and expressing α-amylase gene leads to a high power density of 279.04 mW m^−2^. Since the bacterium *R. anhuiense* belongs to the class of exoelectrogens, it was assumed that *R. anhuiense* could act as the main biocatalyst in an anode compartment to provide electrons and thus to generate electric power. *R. anhuiense* is known to be a bacterium that can survive under oxygen-containing or oxygen-free conditions (in cases when the bacteria are in symbiosis with legume plants). Furthermore, this advantage provides a reason to predict that this nitrogen-fixing bacteria could be used in both biofuel cell compartments (anode and cathode). Since this bacterium could be prescribed to the class of exoelectrogens, further investigations are required to show their capability to act as the main biocatalyst in MFCs.

In this study, we have investigated the applicability of *R. anhuiense* bacteria as the main biocatalyst for constructing dual-chamber microbial fuel cells (MFCs). The carbon felt (CF) anode, used herein as biofilm-hosting electrode, was modified in acidic solutions to provide greater hydrophilicity and improved wetting properties. The bacterial growth kinetics, open-circuit potential variations, and power generation of the designed MFCs have been investigated. Besides, several soluble redox mediators, in particular menadione (MD), riboflavin (RF), and methylene blue (MB), have been applied to enhance the electron transfer from bacteria to solid electrodes.

## 2. Materials and Methods

### 2.1. Materials

All reagents and carbon-based materials in this study were used as received without additional modification, unless otherwise stated. D-Glucose (C_6_H_12_O_6_ 99.5%), 2-methyl-1,4-naphthoquinone (menadione, C_11_H_8_O_2_, 98%), riboflavin (C_17_H_20_N_4_O_6_, 98%), methylene blue (C_16_H_18_ClN_3_S, 96%) dipotassium hydrogen phosphate (K_2_HPO_4_, 98%), magnesium sulphate (MgSO_4_, 99%), calcium carbonate (CaCO_3_, 99%), and sodium chloride (NaCl, 99%) were purchased from Alfa Aesar. Sodium molybdate (Na_2_MoO_4_, 99%), iron sulphate (FeSO_4_, 99%), carbon felt (3.18 mm thick, C, 99%), yeast extract (99%), agar (99%), potassium ferricyanide (K_3_[Fe(CN)_6_], 99%), and proton exchange membrane (PEM) Nafion 115 (125 μm thick) were provided by Sigma-Aldrich Chemical Co. Menadione (MD) was dissolved in 96% ethanol (C_2_H_5_OH, 96%) supplied by Vilniaus Degtine (Lithuania). Milli-Q water (18 MΩ·cm) was used to prepare bacterial cultivation medium, wash microbial reactors, and rinse CF electrode surfaces.

Gram-negative, nitrogen-fixing *Rhizobium anhuiense* bacteria were obtained from Lithuanian Research Centre for Agriculture and Forestry (Akademija, Lithuania) collection of microbial strains. Microorganisms were cultivated in modified Norris media commonly used to cultivate nitrogen-fixing bacteria strains [37]. Notably, in some cases, where the bacteria growth kinetics were estimated, the autoclaved sterile Norris medium was filtered due to the presence of white precipitate mainly caused by the calcium carbonate that is practically insoluble in aqueous solutions. In order to increase the conductivity of the Norris medium, the mixture was supplemented with some additional salts and components that improved the growth rate of *R. anhuiense*. The final composition used for soil bacteria propagation and cultivation is summarized below: norris agar (10 g L^−1^ glucose, 1 g L^−1^ dipotassium hydrogen phosphate, 1 g L^−1^ calcium carbonate, 0.2 g L^−1^ sodium chloride, 5 mg L^−1^ sodium molybdate, 0.2 g L^−1^ magnesium sulphate, 0.1 g L^−1^ iron sulphate, 1 g L^−1^ yeast extract, and 25 g L^−1^ agar), Norris medium (10 g L^−1^ glucose, 0.53 g L^−1^ dipotassium hydrogen phosphate, 6.43 g L^−1^ potassium dihydrogen phosphate 1 g L^−1^ calcium carbonate, 7.48 g L^−1^ sodium chloride, 5 mg L^−1^ sodium molybdate, 0.2 g L^−1^ magnesium sulphate, 0.1 g L^−1^ iron sulphate, and 1 g L^−1^ yeast extract).

### 2.2. Cultivation of R. anhuiense Bacteria

Prior to use, the *R. anhuiense* bacteria was synchronically reinoculated on inclined Norris agar medium and left to grow at 28 °C for 48 h to keep the bacteria fresh. Afterwards, sterile 0.9% sodium chloride solution was filled in the test tubes with inoculums and carefully suspended with an inoculation needle. It should be noted that the harvested culture was looked like small ‘jelly pieces’ in the first 5–10 min. The homogenous bacterial suspension was obtained by vortexing the test tubes for at least five minutes. Then, the bacteria suspensions were transferred and diluted in sterile Norris medium to yield a density of colony-forming units (CFU) equal to 1 × 10^7^ CFU mL^−1^. The bacteria count was established by measuring the optical density of suspension at 600 nm (OD_600_), which was adjusted to be in the range of 0.15–0.2, which corresponds to ~2 × 10^7^ CFU mL^−1^ [38]. The inoculated suspension was left to grow for 24 h at room temperature, with shaking at 160 RPM to achieve the stationary phase (OD_600_ reached about 1.0). The prepared bacterial cells were then used for MFC operations by diluting them ten times with modified Norris medium (pH = 7). The inoculated solutions were cultivated in 50 mL cylinder-shaped Falcon tubes under gentle stirring to investigate the bacterial growth kinetics. The variation in bacteria cell numbers was evaluated by measuring the optical density of the growth media at 600 nm (OD_600_). The concentration of bacteria, usually described in colony-forming units per millilitre (CFU mL^−1^), can be calculated according to the previously reported value, where OD_600_ ≈ 1.0 corresponds to the 1 × 10^8^ CFU ml^−1^ [1]. It was observed that the shape of the cultivation vessel plays an essential role in *R. anhuiense* growth, and this can be associated with different nutrient diffusion rates in the solution. Since the MFC reactor used in this study was H-shaped, we thought that the control investigations of bacteria growth kinetics needed to be conducted in identical conditions. The growth rate of *R. anhuiense* bacteria was evaluated by measuring the OD_600_ for at least 160 h. To investigate the impact of menadione (MD) on the bacteria growth kinetics, 50 and 100 μM of MD (dissolved in ethanol) was added into the cultivation medium before the inoculation. Subsequently, 200 μL of 10 and 20 mM of MD solution was added to the 40 mL of modified Norris medium prior to inoculation with bacteria. All measurements were performed in triplicate for each sample. The control measurements with ethanol were conducted and acted as a negative control. MD is a lipophilic redox mediator that can freely penetrate through the living cell membrane and interact with intracellular redox species such as mitochondrial or cytosolic enzymes [39,40]. However, higher dosage of this compound leads to the generation of reactive oxygen species (ROS) associated with mitochondrial DNA damage that causes cell death [41]. This feature encouraged us to investigate the antimicrobial activity of MD on the *R. anhuiense* bacteria growth.

In order to investigate the morphology of *R. anhuience*, the optical images were acquired by using Olympus BX51 Fluorescence Phase Contrast Microscope (Japan) and an oil immersion technique. For this purpose, the bacterial samples were taken from a freshly grown suspension and washed three times with 0.9% sodium chloride solution prior to optical microscopy analysis.

The average cell length and cell length distribution were estimated by measuring at least 50 cells from optical images using ImageJ (USA) software.

### 2.3. Preparation of Carbon Felt-Based Electrodes

As received, carbon felt (CF) was cut into 20 × 40 and 20 × 80 mm pieces and further cleaned following the procedure reported previously [42]. Briefly, the electrodes were ultrasonically cleaned in ethanol and deionized (DI) water for 480 s, with the procedures repeated three times to eliminate possible organic impurities. Subsequently, the cleaned CF specimens were dried by airflow and transferred to the acidic oxidation bath filled with concentrated 3:1 (*v*/*v*) sulphuric acid and nitric acid at a temperature ranging from 35 °C to 45 °C. The reaction was conducted for 4 h under vigorous stirring. This process is frequently used for carbon-based materials, such as carbon fibres, cloth, nanotubes etc., the modification was the formation of the oxygen-containing groups on the surface of carbon, enabling the greater hydrophilicity and improving wetting properties of the material [43]. Moreover, surfaces with enhanced wetting properties are considered to be more suitable for biomolecules, enzymes or even microorganisms’ immobilization [44,45]. After draining of the acidic mixture, the modified CF electrodes were washed with dozens of DI water until a neutral pH was reached for the washing solution. The obtained electrodes were dried in airflow and heated in a muffle furnace at 120 °C for 5 h to evaporate the water completely.

### 2.4. Microbial Fuel Cell Set-Up

**Figure 1:**
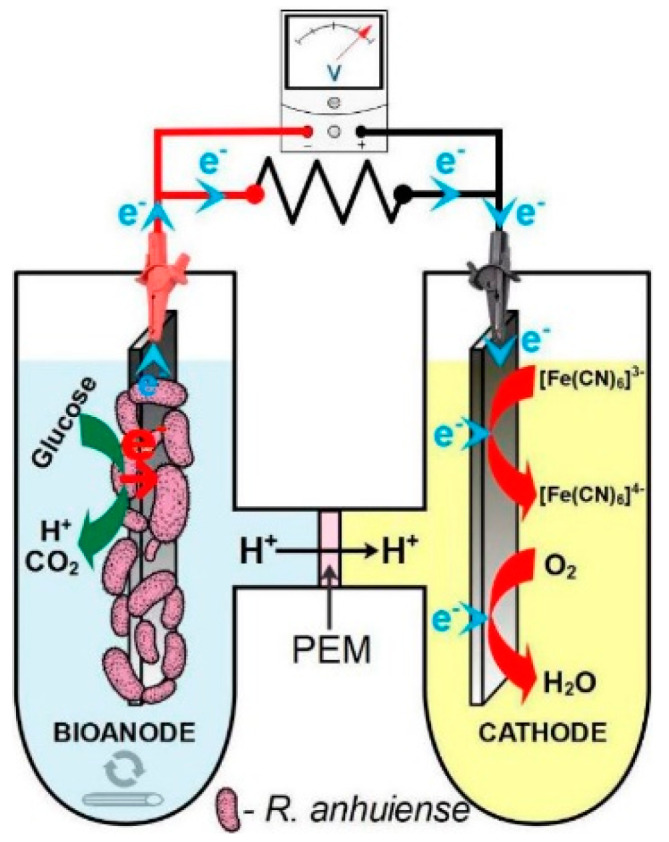
Schematic illustration of H-type, dual-chamber *R. anhuiense*-based microbial fuel cell used in this study.

The *R. anhuiense*-based microbial fuel cell (MFC) setup consisted of two cylindrical-shaped chambers each filled with 40 mL of working solution and connected by ~10 mm in diameter tube as presented in Figure 1. The anode and cathode compartment were separated by Nafion 115 membrane utilized herein as the proton exchange membrane (PEM) with proton exchange capacity of ≥0.90 meq/g and surface area of 0.785 cm^2^. Since the distance between anode and cathode plays an important role in MFC performance [46], in this study, it was set to be no more than 20 mm, which was expected to reduce the energy losses related to the decreased ohmic resistance of the system. Each reactor contained one modified carbon felt anode and a cathode with a two-fold higher geometric surface area. The anodic chamber was filled with modified Norris medium inoculated with *R. anhuiense* bacteria, ensuring that the final concentration of bacteria cells expressed in CFU mL^−1^ was approximately ~10^7^. It is essential that the anode chamber is continuously stirred; otherwise, a higher amount of bacteria will precipitate much more quickly than other commonly known bacterial strains, such as *P. aeruginosa*, *M. luteus*, etc., and obviously cannot act as a biocatalyst.

The inoculated medium was stirred at 200 RPM throughout the MFC lifetime, except when the power density measurements were conducted, to avoid the bacteria sedimentation. Furthermore, several membrane-permeable redox mediators, such as menadione (MD), riboflavin (RF), and methylene blue (MB), were utilized in this study to enhance the MFC performances. Following this approach, each compound with a concentration ranging from 1 to 50 μM was added into the anode compartment when the steady-state potential difference between the anode and cathode was achieved. Meanwhile, the cathodic chamber was fed with phosphate-buffered saline (PBS) solution containing 40 mM of potassium ferricyanide used herein as an electron acceptor. Notably, the prepared MFCs were operated at ambient temperature. Following the protocol highlighted recently by Logan et al. [47], the MFCs were acclimatized by connecting both electrodes with 100 Ω external resistance (R_ext_) for 5 h on the first two days to enhance the performance of MFCs.

### 2.5. Electrochemical Characterization of MFC

The electrochemical characterization of *R. anhuiense*-based MFC was performed using a Zahner Zennium electrochemical workstation (Zahner-Elektrik, Germany) and Thales XT software. For each particular electrode, the time-dependent open-circuit potential (OCP) was estimated by using a digital multimeter and double junction Ag/AgCl reference electrode (standard potential vs. saturated hydrogen electrode (SHE) was +205 mV) filled with 3 M KCl (Metrohm, Switzerland). The whole-cell OCP was calculated following the equation *OCP* = (*E_k_* − *E_ref_*) − (*E_a_* − *E_ref_*), where *E_k_* is the potential cathode and *E_a_* is the anode potential. The OCP value of the biofilm-hosting electrode (bioanode) usually becomes more negative due to the multiple half-reactions that occur at the electrode/solution interface and eventually approaches the thermodynamic limit for substrate oxidation [48]. The open-circuit potential of electrodes depends on various redox-active species located at the electrode surface. Membrane-bound redox protein such as respiratory electron transport chains, for instance c type cytochromes, are the most likely to react if the microorganisms are capable of communicating with solid electrodes via a direct electron transfer (DET) mechanism [49]. The other option, which impacts OCP value, is the soluble redox mediators, such as phenazines, pyocyanin, and others that can be secreted by bacteria itself [50]. Ultimately, the electrolyte composition and pH play an essential role in equilibrated electrode potential in the solution, especially when the oxygen and soluble redox mediators are presented to the working electrolyte. In order to separate possible interference half-reactions, the control measurements need to be conducted when measuring the OCP potential through the extended period.

The polarization curves of designed MFCs reactors were recorded using the linear sweep voltammetry (LSV) method by sweeping the potential from OCP (the negative value of the potential difference between anode and cathode) to 0 mV at 0.1 mV s^−1^. These measurements were achieved by connecting the biofilm-hosting electrode (anode) to the working electrode and the CF cathode to the combined reference and counter electrode in a dual-chamber, H-type MFC set-up. The power density of MFC was calculated according to equation P = U·I/S_surf_ by multiplying voltage by current values (obtained from polarization curve measurements) and dividing by the geometric surface area. In order to investigate the possible interference reactions that occur on the CF electrode in the potential window relative to the MFC operating potential, cyclic voltammetry (CV) analysis was performed with each electrode separately in modified Norris medium and PBS containing 40 mM of potassium ferricyanide solutions. CV scans were recorded at the potential scan rate of 10 mV s^−1^ in a three-electrode configuration cell, where the CF electrode acted as a working electrode and the Ag/AgCl and platinum plate acted as a reference and auxiliary electrodes, respectively. The electrolyte solutions were extensively saturated by bubbling N_2_ for 2 h to determine the influence of oxygen and keep it above the solution during the measurements.

## 3. Results

### 3.1. Evaluation of R. anhuiense Bacteria Morphology and Cells Growth Kinetics

**Figure 2:**
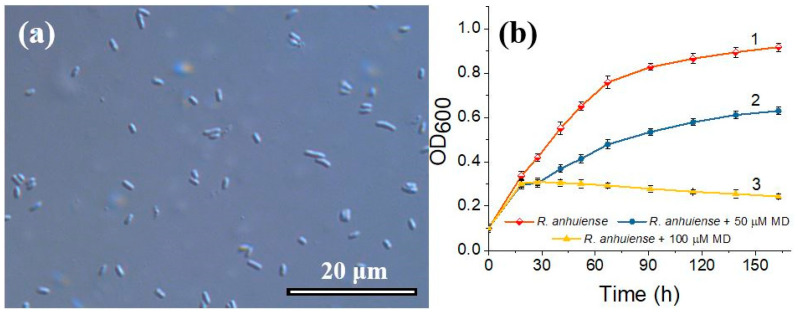
Optical microscope image of *R. anhuiense* bacteria cells (**a**) and their growth kinetic curves (**b**) obtained by measuring the absorbance of the inoculated Norris medium incubated without (curve 1) and with the presence of 50 (curve 2) and 100 μM (curve 3) of menadione. All bacterial suspensions were cultivated in a shaking incubator at 160 RPM under ambient conditions. Error bars represent the standard deviation (SD) of OD_600_.

The morphology of *R. anhuiense* was tested to investigate the size and shape of the bacteria used in this study as the main biocatalyst for designed *R. anhuiense*-based MFC. From the images obtained via optical microscope and presented in Figure 2a, it was evident that *R. anhuiense* bacteria are rod-shaped with cell lengths varying from 1.4 to 2.6 μm. The average cell length and its distribution, estimated by measuring at least 50 cells from optical images, was determined to be approximately 2.15 μm.

In this study, a nitrogen-fixing bacteria strain named *R. anhuiense* was cultivated in modified Norris medium at ambient conditions (20 ± 1 °C) to examine the impact of MD on bacteria growth. The results showed that the growth rate of *R. anhuiense* was increased by approximately 1.6 times if the inoculate was cultivated at 28 °C (data not presented). An ambient temperature regime was chosen in this study because *R. anhuiense* is a nitrogen-fixing bacteria prevalent in soil and grows under environmental conditions. According to the growth curves presented in Figure 2b, it is obvious that the highest *R. anhuiense* proliferation rate is observed in the exponential cell growth phase at the time period from 0 to 67 h (curve 1). Further cultivation of this bacteria resulted in a slower growth rate, which eventually reached the stationary phase, possibly due to the consumption of nutrients, while the obtained OD_600_ variation over a 96-h period was only 0.159 (Figure 2b, curve 1).

The obtained results showed that the presence of 100 μM of MD was disastrous for *R. anhuiense* bacteria growth through long-term cultivation (Figure 2b, curve 3), in which the OD_600_ of the solution started to decrease at 18.5 h after the inoculation. However, in the case of 50 µM MD, the viability of the bacteria cells was less affected, the decrease in OD_600_ compared with the control aliquot was approximately 32–37% (Figure 2b, curve 2). These results confirmed that MD could be utilized as a redox mediator for *R. anhuiense* at lower concentrations (less than 50 μM), whereas the bacteria remain viable and can successfully proliferate, even at ambient conditions. It should be noticed neither ethanol (0.48%, *v*/*v*) nor 5 μM of MD significantly impacted the bacterial growth curve and overlapped with curve 1, presented in Figure 2b.

### 3.2. Open-Circuit Potential Investigations of MFC

**Figure 3:**
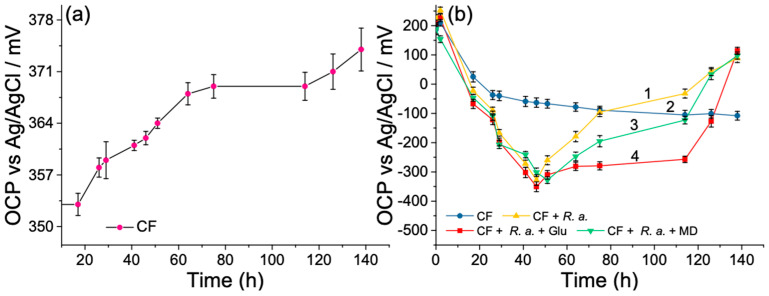
Time-dependent open-circuit potential variations of bare CF cathode (**a**) and various CF anodes (**b**) estimated in the modified Norris media (pH = 7.0) without (curve 2) and with inoculums of *R. anhuiense* bacteria (1 curve). Curves 3 and 4 correspond to the OCP changes measured in similar solutions enriched with 5 μM MD (curve 3) and 12.5 mM glucose after 51, 75, and 114 h, respectively (curve 4). All error bars denote the standard deviation (SD) of OCP.

In this study, the initial OCP value of CF anode was positive in the 187–224 mV range, as presented in Figure 3b, curves 1–4. After the inoculation of *R. anhuiense* bacteria cells (10^7^ CFU mL^−1^) to the anode compartment, the OCP gradually drifted to the negative direction and reached maximum values in the range of −301 to −351 mV after cultivation for 41 h in ambient conditions. These results validated the existence of electroactive bacteria that potentially formed a biofilm on CF electrode surfaces. Compared with the control measurements (Figure 3b, curve 2), the potential difference caused by *R. anhuiense* bacteria at its maximal value differed from the solution over the potential range from −238 to −288 mV (Figure 3b, curves 1, 3, 4). It should be highlighted that the presence of 5 μM MD did not significantly affect the OCP curve of the bioanode in the first part of the experiment; however, in the time range from 51 to 138 h, the reduction of the potential (becoming less positive) became gradually larger in comparison with the control (Figure 3b, curve 3). It was assumed that the main reasons for this potential decrease could be related to the depletion of nutrients in the bacterial growth medium, as clarified in previous studies [51]. This hypothesis can be confirmed by comparing the OCP curves with the bacterial growth kinetics, whereas the time required to achieve the steady-state phase of bacteria growth was well matched to the time relative to the starting OCP reduction point. Furthermore, the addition of 12.5 mM of glucose after 51 and 75 h of cultivation kept the electrode potential more negative than others, evidencing the requirement for carbon substrate renewal in the electrolyte solutions (Figure 3b, curve 4). However, the third addition of glucose (after 114 h from starting point) did not significantly impact the bioanode potential and continued to decrease by reaching the saturated values that fitted in the gap from 89 to 114 mV.

The time-dependent CF cathode potential variations have been investigated to examine the stability of catholyte solutions through the long-time investigations of MFCs. For this purpose, the CF electrode was immersed into a PBS solution containing 40 mM of potassium ferricyanide, which was used in this study as an electron acceptor. When comparing the cathode OCP shifts during the operating time of MFCs with the bioanode on the same graph, the first one seems to be a straight line. Despite this, some fluctuations were observed over the 138-h continuous measurements (Figure 3a). The OCP value of the CF electrode slowly increased from 353 to 374 mV in almost six days lasting measurements. This phenomenon was mainly related to the stability of K_3_[Fe(CN)_6_] complex, which can be decomposed by light and the molecular oxygen that is dissolved in aqueous solutions [52]. However, variations in ΔOCP as high as 21 mV over 138 h did not significantly impact the MFC operation and characterization.

### 3.3. Power Output of Dual-Chamber MFC

**Figure 4:**
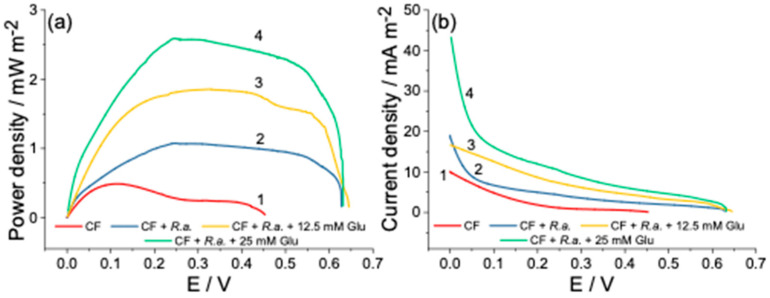
Power density (**a**) and polarization curves (**b**) of mediator-less MFCs recorded by using the LSV method in modified Norris medium at the potential sweep rate of 0.1 mV s^−1^ in two-electrode configuration mode, whereas the biofilm hosting electrode (bioanode) was connected to the working electrode and the cathode to the combined counter-reference electrode, respectively. Electrochemical analysis was performed without (curve 1) and with the presence of *R. anhuiense* bacteria (curves 2–4). Prior to measurements (at least 5 h), 12.5 and 25 mM of glucose were added to the anode chamber (curves 3 and 4), respectively.

The potential differences estimated herein as a separated CF electrode (anode and cathode) potential referred to the Ag/AgCl_3M KCl_ reference electrode determines the whole MFC potential, which ranged from 627 to 644 mV, as can be seen in Figure 4a,b. The polarization and power density curves recorded under the given conditions are displayed below and possess a typical shape of an MFC power plot reported elsewhere [53]. In order to verify the bacteria electrogenicity, the control measurement was conducted, in which the anode chamber was left without the bacterial inoculation. Interference reactions, in particular the oxygen reduction reaction (ORR) and the reduction of Fe^3+^ to Fe^2+^, are unavoidable since the MFCs were operated under aerobic conditions by using the catholyte, which contains K_3_[Fe(CN)_6_], utilized in this study as an electron acceptor. The obtained results showed the power generated by the control aliquot that could mainly be attributed to the abovementioned process, which spontaneously occurs at the electrode/solution interface at the potential window tested herein (Figure 4a, curve 1). However, the power density curve’s profile changed dramatically after inoculating the anodic chamber with *R. anhuiense* bacteria and subsequently achieved a maximal MFC power output of 1.077 mW m^−2^ at an operating voltage of 245 mV (Figure 4a, curve 2). As described above, when the substrate was re-fed in the anode compartment, the bioanode potential was kept at negative values for much longer, and a similar dependency was obtained here, in which the glucose addition positively impacted the power generation produced by MFC. It should be noticed that the polarization curves were recorded at least 5 h after the addition of substrate to achieve the equilibrium stage. As shown, after the first addition of 12.5 mM lucosee (after 51 h), the power density of MFC increased by over 72% and reached 1.862 mW m^−2^ at an operating voltage of 327 mV (Figure 4a, curve 3).

**Figure 5:**
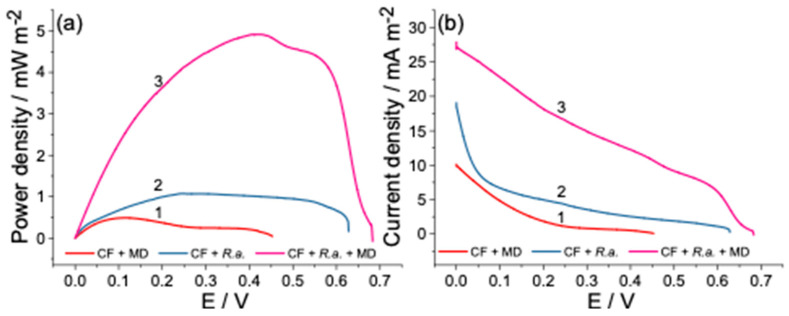
Power density (**a**) and polarization curves (**b**) of *R. anhuiense*-based MFCs recorded in modified Norris medium at the potential sweep rate of 0.1 mV s^−1^ in two-electrode configuration mode. Electrochemical tests were performed without (curve 1) and with *R. anhuiense* bacteria (curves 2 and 3). Prior to measurements (at least 5 h), 5 μM of menadione (MD) was added to the anode chamber (curve 3).

Furthermore, the second addition of identical amounts of glucose increased the power density values up to 2.585 mW m^−2^, which was over ~240% higher than the control aliquot and registered a potential of 281 mV (Figure 4 a, curve 4). By comparing the power density curves (2–4), it can be summarized that the substrate addition is necessary to increase the *R. anhuiense*-based MFC performance when an H-type cell design is used. The current density registered at the beginning of the measurement increased from 10.0 mA m^−2^ (without *R. anhuiense* bacteria) to 43.3 mA m^−2^ (in the presence of 25 mM of glucose) as displayed in Figure 4b, curves (1 and 4). The established power density generated by the *R. anhuiense*-based MFC device complies with other researchers’ proposed MFC reactor power outputs summarized in Table 2. According to the obtained results, only the addition of menadione (from all tested redox mediators utilized herein) has a positive impact on MFC-generated power density. Thus, the latter was further investigated with a concentration ranging from 1 to 50 μM. It should be considered that MD can be toxic for bacteria cells at higher concentration ranges, as evidenced by estimating the variations of bacterial growth. The optimal concentration of MD was found by measuring the series power output of MFC provided at its maximal OCP. The results obtained from these measurements showed a concentration-dependent increase in power density from 1 to 14 μM (data not presented) and displayed an exponential curve profile. However, this increase follows linear regression only from 3 to 9 μM; thus, in accordance with other research [54], 5 μM of MD was set to be an optimal concentration of mediator that did not influence the growth of the microorganisms, as confirmed above. The MFC exhibited the best performances in the presence of MD while the power generated by this device was 9.6 times higher than the control aliquots and reached a power density of 4.93 mW m^−2^ at an operating voltage of 419 mV, as can be seen in Figure 5a, curves 1 and 3.

### 3.4. Investigation of Interference Reactions

**Figure 6:**
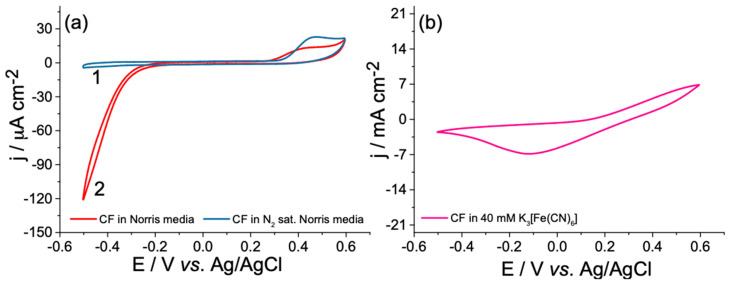
Cyclic voltammograms of bare CF anode (**a**) recorded in N_2_ (curve 1) and air-saturated (curve 2) modified Norris medium (pH = 7.0) and cathode (**b**) analyzed in PBS catholyte (pH = 7.0) containing 40 mM of potassium ferricyanide in the potential window of 0.6 to −0.5 V at a scan rate of 10 mV s^−1^.

Since the designed MFC was operated under aerobic conditions in a wide potential window, the possible interference reactions that can occur spontaneously at the given potential diapason deserved to be clarified herein. For each particular electrode (bioanode and cathode), the potential differences between electrodes at open circuit conditions were determined, resulting in its average values ranging from −326 to 363.5 mV vs. Ag/AgCl_3M KCl_. The oxygen reduction reaction (ORR) that occurs on CF in negative potential ranges with the onset potential of −189 mV was evidenced by bubbling nitrogen gas through the electrolyte solutions for 2 h. This process resulted in a significant current decrease by proving its origin in the ORR (Figure 6a, curves 1–2).

On the opposite side, the CV analysis observed the oxidation wave with an onset potential of 309 mV. It was assumed that this irreversible oxidation process could be attributed to the oxidation of some unknown organic compounds that can be found in yeast extract. The CF working electrode was also electrochemically investigated by cyclic voltammetry in the electrolyte with an identical composition to the catholyte, which contained 40 mM of K_3_[Fe(CN)_6_]. As shown in Figure 6b, the wide reduction wave of Fe^3+^ to Fe^2+^ was observed with a peak potential (*E_pc_*) value of −119 mV. It could be summarized that the interference reactions at bioanode take place only at the ends of the MFC operating potential window, and could thus be ignored when estimating the performances of MFC. Besides, the current density delivered by MFC is limited by the reaction rate at one particular electrode surface [64]. From this point, the anodic current densities of similar-sized CF electrodes are significantly higher than the opposite ones (as evident when comparing the current density values in Figure 6a,b), thus making the anode the limiting part of the designed MFC.

## 4. Discussion

**Figure 7:**
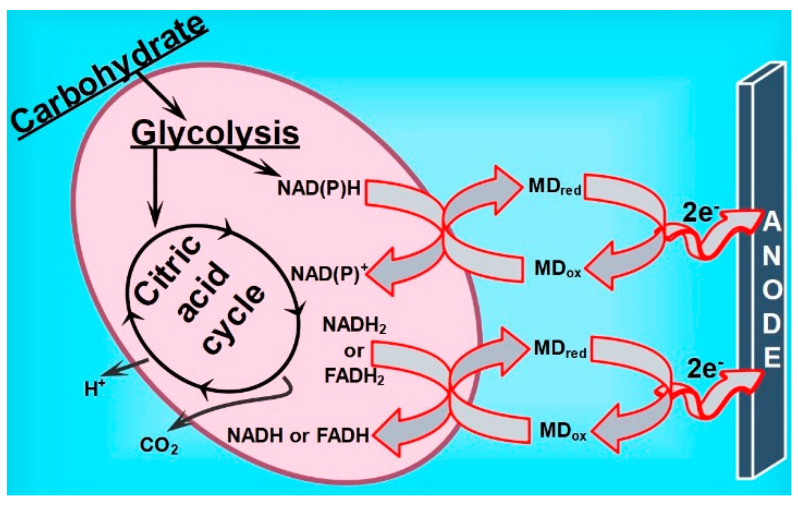
Schematic illustration of MD-mediated electron transfer mechanism from the *R. anhuiense* bacteria metabolism to the CF anode.

The efficiency of MFC depends on various aspects, including cell design, the electrodes used, and the biocatalyst, but mainly on the charge transfer efficiency towards conductive surfaces, which usually determine the whole device performances [65]. Bacteria cells are adapted to use various organic compounds, including carbohydrates, lipids, and proteins, as the main carbon sources. These organic nutrients act as electron donors for many complex redox reactions; thus, molecules of the energy carrier adenosine triphosphate (ATP) have been produced. Depending on the main carbon sources, the nutrients can be metabolized by bacteria through glycolysis and related processes into acetyl-CoA molecules, and further subjected to the citric acid cycle, as shown in the scheme in Figure 7. At this stage, the redox reaction is coupled to the reduction of NAD^+^ and FAD to their oxidized/reduced forms (NADH and FADH_2_) [66]. In these cases, where the bacteria are weak exoelectrogens, the soluble redox shuttle that carries electrons to the solid electrodes is required to enhance or even detect the current densities provided by MFC [67]. Both forms of MD (oxidized and reduced) are neutral and lipophilic, with the molecular structure close to ubiquinone known as a membrane-bound redox mediator [68]. The electron transfer mechanism in such systems is mainly based on its permeation through the cell outer membrane and reduction by the redox enzymes to menadiol (MD_red_) that are located in the cytosol or mitochondria and catalyzing the electron transfer from NAD(P)H to quinone substrates [63]. The MD_red_ further diffuses outside the bacteria cell and interacts with the CF electrode, being oxidizing to the previous form of MD_ox_ and completing the cycle as illustrated in the schematic in Figure 7. Based on the power outputs generated by the MFC device in this study and by comparing them with previous research (see Table 2), it can be assumed that gram-negative *R. anhuiense* bacteria cannot be prescribed to the class of strong exoelectrogens. However, it was found that the menadione redox mediator could cause a 10-fold increase in MFC performance. Nevertheless, the obtained power density value (4.93 mW m^−2^) dictated that the electron transfer rate between *R. anhuiense* and CF electrode was not sufficient in comparison with today’s most powerful MFC devices, where the values of their power output range from several hundred to a few Watts per square meter [69]. It was assumed that an electron acceptor—molecular oxygen—could take a significant amount of electrons, making the whole device less efficient.

To the best of our knowledge, the nitrogen-fixing *R. anhuiense* bacterium has never been used as a main biocatalyst in MFC devices. The obtained energy output values seem to be promising, boosted by the fact that these microorganisms naturally grow in aerobic conditions except the stage when participating in symbiosis with legume plants on their roots [70]. Although there is minimal information about the biochemical structure and possible electron transfer chains inside *R. anhuiense* cells, it was shown that by using menadione as a redox mediator, this soil bacterium could be successfully used as a main biocatalyst for the construction of MFC.

## 5. Conclusions

Here, we have shown that the nitrogen-fixing, Gram-negative bacterium *R. anhuiense* could be successfully utilized as a main biocatalyst in the anode compartment by using modified carbon felt anode in an H-type microbial fuel cell setup. Our results demonstrated that *R. anhuiense*-based MFC performances could be enhanced by over 240% compared to control by re-feeding the anode bath with glucose after cultivation for 75 h. Moreover, the corresponding anode potential and power density can be improved if 5 μM menadione was added to the modified Norris medium. Notably, this amount of redox mediator does not significantly impact *R. anhuiense* bacteria growth; thus, it can be used without any side effects. In this case, the designed MFC’s maximal open-circuit voltage and power density were estimated to be 683 mV and 4.93 mW m^−2^, respectively. Overall, this research opens a new avenue for the *R. anhuiense* bacteria to be exploited as a main biocatalyst in bacteria-based MFCs.

## Figures and Tables

**Table 1:** List of top bacteria reported as exoelectrogens.

Reported List of Exoelectrogens	References
Firmicutes, Proteobacteria, Acidobacteria, fungi, and algae	[19]
Proteobacteria, Bacteroidetes, Chloroflexi, Acidobacteria, Firmicutes, and Nitrospirae	[31]
*Geobacter psychrophilus*, *Pseudomonas caeni*, *Simplicispira sychrophile*, *Comamonas badia*, and *Geobacter chapelle*	[32]
*Clostridium butyricum*, *Rhodoferax ferrireducens*, *Shewanella* sp., *Geobacter* spp., and *Aeromonas hydrophila*	[28]
*Geobacter sulfurreducens*	[24]
*Natronocella acetinitrilica*, *Beijerinckiaceae*, *Rhizobiales*, and *Rhodobacter gluconicum*	[33]

**Table 2:** Comparison of performances of various yeast-based and bacteria-based MFCs.

MFC Set-Up	Anode Compartment	Cathode Compartment	Carbon Source	Open Circuit Voltage (OCV), mV	Maximal Power Output (*P_max_*), mW m^−2^	Reference
H-type cell	Graphite felt/*P. aeruginosa* (pilT) mutant	Pt/carbon cloth + 50 mM of K_3_[Fe(CN)_6_]	Urea	720	54.16	[55]
	Graphite felt/*P. aeruginosa* (wild-type)			750	20.0	
Batch-type	Graphite carbon cloth/*E. coli* (gene type 1) + 11.5 mM of methylene blue	Graphite carbon cloth + 0.1 M of K_3_[Fe(CN)_6_]	Beef extract; peptone.	309 *	21.7 *	[56]
	Graphite carbon cloth/*E. coli* (gene type 2) + 11.5 mM of methylene blue			470 **	8.36 **	
	Graphite carbon cloth/*E. coli* (gene type 3) + 11.5 mM of methylene blue		Yeast extract; tryptone	570 ***	134 ***	
Single chamber, air-cathode	Carbon paper/*S. cerevisiae*	Carbon paper with Pt catalyst (1 mg cm^−2^)	Glucose	550	3.0	[57]
Single chamber, open-air cathode	Gold-sputtered carbon paper/*S. cerevisiae +* 20 g L^−1^ of yeast extract	Carbon paper with Pt catalyst (1 mg cm^−2^)	Glucose; Peptone	910	70.0	[58]
Batch-type	Carbon paper/*G. sulfurreducens* + 50 mM of fumarate	Carbon paper	Acetate	-	16.2	[59]
Single-chamber, air-cathode	Graphite block modified by graphene/*S. oneidensis*	Carbon paper with Pt catalyst (0.5 mg cm^−2^)	Sodium lactate	780	102	[60]
Dual chamber	Carbon felt/*Cystobasidium slooffiae*	Carbon felt + 50 mM of K_3_[Fe(CN)_6_]	Xylose	540	67	[61]
Single-chamber, open-air cathode	Au-sputtered carbon paper + *S. cerevisiae*	Carbon paper with Pt catalyst (1 mg cm^−2^)	Glucose	600	2	[62]
Dual chamber	Stainless-steel/*L. starkeyi*	Stainless-steel + KMnO_4_	Effluent	900	47.6	[63]
	Stainless-steel/*K. pneumonia*			800	19.77	
	Stainless-steel/co-culture of *L. starkeyi* and *K. pneumonia*			750	10.98	
H-type cell	Carbon felt/*R. anhuiense*	Bare carbon felt + 40 of mM K_3_[Fe(CN)_6_]	Glucose	635	2.59	This work
	Carbon felt/*R. anhuiense* + 5 μM of menadione			683	4.93	

* After 18 h of cultivation; ** after 16 h of cultivation; *** after 22 h of cultivation.

## Data Availability

Not applicable.
